# Identification of the First Case of Dedifferentiated Liposarcoma in Amphibians: Insights from *Maculopaa medogensis*

**DOI:** 10.3390/vetsci11110526

**Published:** 2024-10-30

**Authors:** Runliang Zhai, Tianyu Qian, Yonghong Wang, Bin Wang, Cheng Li, Feng Xie, Wei Zhu, Jianping Jiang

**Affiliations:** 1 Chengdu Institute of Biology, Chinese Academy of Sciences, Chengdu 610213, China; zhairunliang22@mails.ucas.ac.cn (R.Z.); qianty@cib.ac.cn (T.Q.); wangyongh0928@163.com (Y.W.); wangbin@cib.ac.cn (B.W.); licheng@cib.ac.cn (C.L.); xiefeng@cib.ac.cn (F.X.); 2 University of Chinese Academy of Sciences, Beijing 10049, China; 3 Mangkang Biodiversity and Ecological Station, Tibet Ecological Safety Monitor Network, Changdu 854500, China

**Keywords:** tumor, amphibian, micro-CT, histology, immunohistochemistry

## Abstract

Dedifferentiated liposarcoma (DDLPS) is a rare and aggressive type of cancer primarily reported in humans, with no documented cases in animals. In this study, we present the first case of DDLPS in a wild amphibian species, *Maculopaa medogensis*. The tumor was discovered during a routine examination and diagnosed through a combination of advanced diagnostic methods, including micro-CT imaging, gross anatomical inspection, histological analysis, and immunohistochemistry. The tumor exhibited both well-differentiated and dedifferentiated components, characteristic of DDLPS, with evidence of tissue invasion and multiple metastases. Immunohistochemical analysis revealed the strong positive expression of markers such as S100A4, CDK4, MDM2, and CD34, while Leptin expression was negative, further confirming the diagnosis. This is the first reported case of DDLPS in a non-human species, expanding our understanding of cancer in wildlife and underscoring the significance of amphibians as environmental indicators. These findings provide valuable insights for veterinary medicine and wildlife conservation, particularly regarding the role of environmental stressors in cancer development. This study highlights the need for comprehensive diagnostic approaches in wildlife pathology.

## Simple Summary

Dedifferentiated liposarcoma (DDLPS) is a rare and aggressive type of cancer that originates in fat tissue and has previously only been reported in humans. In this study, we document the first case of DDLPS in a wild amphibian species, *Maculopaa medogensis*. Using a combination of advanced diagnostic techniques, including micro-CT scanning, histological analysis, and immunohistochemistry, we confirmed the diagnosis. This case provides important insights into the development of DDLPS in non-human species, highlighting its potential links to environmental stressors. Amphibians, as environmental indicators, offer a unique perspective on how changes in ecosystems may contribute to the emergence of diseases like cancer. Our findings emphasize the importance of comprehensive diagnostic approaches in veterinary pathology and contribute to the growing body of research on wildlife health. Studying cancers across species helps to improve both veterinary and human medicine, offering new perspectives on cancer biology and potential treatments.

## 1. Introduction

In recent years, research on amphibian tumors, though relatively limited, has garnered increasing attention. By 1986, there had been 491 documented cases of spontaneous neoplasms in anurans and approximately 253 in caudates [1]. Pathological reports describe amphibian tumors such as dermal papillomas and lymphomas, primarily affecting the skin and hematopoietic systems [2,3]. In addition to skin and hematopoietic tumors, soft tissue tumors, such as sarcomas, originating from connective tissues, including muscle and fat, have also been reported [4]. While the incidence of these tumors in amphibians is relatively low, their presence highlights the diversity of neoplastic conditions in these species. Although the pathology of amphibian cancers remains understudied, the documentation of these tumor cases, along with related data, offers valuable insights into amphibian disease patterns and can contribute significantly to biological conservation efforts.

Soft tissue sarcoma is an extremely rare malignant tumor that originates from the mesoderm, commonly affecting connective tissue, muscle, and adipose tissue [5]. The concept of dedifferentiated liposarcoma (DDLPS) was first proposed in 1979 [6]. Initially, it was believed that the dedifferentiated component developed from an atypical lipomatous tumor/well-differentiated liposarcoma (ALT-WDLPS) over time. However, further research has shown that the dedifferentiated component exists independently and does not transform from ALT-WDLPS. This finding suggests that DDLPS can arise as a distinct tumor type rather than as a progression from a less malignant form [7,8].

Histologically, DDLPS presents as areas of well-differentiated fat tissue mixed with poorly differentiated, non-fatty tissue. Immunohistochemical markers, including MDM2 and CDK4, play a key role in diagnosing this subtype of liposarcoma [9]. Previous research demonstrates that MDM2 and CDK4 exhibit sensitivities of 95% and 92%, and specificities of 81% and 95%, respectively, for DDLPS diagnosis. Therefore, immunohistochemical analysis is critical for ensuring accurate diagnosis [10].

Research on liposarcoma in animals is less common than research on human cases, with even fewer reports on subtypes, leading to an imbalance in the understanding of liposarcoma across species [11]. Currently, although soft tissue tumors are common in classic veterinary animals like dogs, there have been no reports clearly classifying them as DDLPSs in animals, highlighting a gap in veterinary medicine.

In this study, we report a case of DDLPS in *Maculopaa medogensis*, diagnosed using micro-CT scanning, gross anatomy, histological examination, and immunohistochemistry. Due to the absence of previous reports on DDLPS in non-human species, this case provides critical insights that can enhance our understanding of cancer in non-human species. Considering the unique evolutionary position of amphibians as a transitional group between aquatic and terrestrial environments, this discovery of DDLPS in *M. medogensis* not only advances our understanding of cancer development in wildlife but also provides critical insights for conservation efforts.

## 2. Materials and Methods

### 2.1. Field Collection and Artificial Conservation

The animal described in this case report was found on 18 October 2023, in a drainage culvert on a winding mountain road in Renqingbeng Township, County Medog, Xizang Autonomous Region. The culvert, an artificial cave with abundant water and water storage capacity, provided a suitable living environment for the animal. Using a portable water quality monitoring device (Smart sensor, Dongguan, China), we measured the water quality at the locations where the animals were observed: pH values ranged from 6.9 to 8.6, conductivity from 0 to 21 μS, dissolved oxygen levels from 10.22 to 14.6 mg/L, and water temperatures from 8.5 to 15.4 °C.

As a widely distributed species in the region (at an altitude of approximately 1500 m), *M. medogensis* was found in multiple culverts during the survey, with 2–6 adult individuals and tadpoles observed in each culvert. Additionally, traces of other amphibian species, including *Jingophrys pachyproctus*, *Rhacophorus burmanus*, and *Polypedates braueri* were detected in and around the culverts. A total of nine individuals of *M. medogensis* were collected from the area, and subsequently transported to the Chengdu Institute of Biology, Chinese Academy of Sciences.

Under artificial conservation conditions, the animal was fed mealworms every other day and housed individually in an enclosure designed to replicate its natural habitat. The enclosure included a water purification system with a pump to simulate running water, as well as rocks covered with moss. Water quality measurements showed pH values ranging from 8.1 to 8.6, conductivity from 543 to 800 μS, dissolved oxygen levels from 8.7 to 13.4 mg/L, and water temperatures from 17.2 to 21.8 °C.

### 2.2. Observation of Tumor Formation and Micro-CT Scanning

On 10 November 2023, we observed a subcutaneous mass on the right dorsal–ventral side of one animal. Initially, the mass was soft to the touch, but it increased in size over time, eventually becoming partially firm. Additionally, the animal exhibited signs of lethargy and reduced activity. Unfortunately, since no physical examination was conducted upon the animal’s transportation to the laboratory for artificial feeding, the exact time of tumor formation cannot be determined.

On 14 December 2023, following ether anesthesia, the frog was sent for CT scanning at the Chengdu Institute of Biology, Chinese Academy of Sciences. A high-resolution X-ray scanner (Quantum GX micro-CT Imaging System, PerkinElmer, Waltham, MA, USA) and RadiAnt DICOM Viewer software (Medixant, Poznań, Poland) were used for CT imaging and analysis. Images were scanned along the coronal axis at a resolution of 2000 × 2000 pixels. Each scan was performed at a voltage of 90 kV, a current of 88 μA, and for 512 projections in 4 min [12]. 

### 2.3. Necropsy, Histopathology, and Immunohistochemistry

On 15 December 2023, following euthanasia with ether, a gross necropsy was performed, and samples were collected for further analysis. The extracted pathological tissue was fixed in 4% paraformaldehyde solution for two days, then sent to Wuhan Servicebio Co. Ltd. for immunohistochemistry (IHC) and hematoxylin and eosin (H&E) staining. 

In the IHC staining procedure, after deparaffinizing the sections, antigen retrieval was performed as specified in Table 1. The slides were then cooled and washed in PBS (pH 7.4). Endogenous peroxidase activity was blocked using 3% hydrogen peroxide, incubated in the dark for 25 min. To reduce non-specific binding, serum blocking was performed using 3% BSA for 30 min at room temperature. The primary antibody, prepared in PBS, was applied and incubated overnight at 4 °C. The following day, the slides were washed and incubated with an HRP-conjugated secondary antibody for 50 min. DAB staining was performed under microscopic observation until the positive areas turned brown. A hematoxylin counterstain was then applied, and after differentiation, the nuclei were restored to blue. Finally, the slides were dehydrated and mounted for analysis. 

Staining results from both tumor and normal tissues were used as positive and negative controls to confirm antibody specificity and validate its ability to distinguish between tumor and normal tissues.

The antibodies used in IHC were S100A4 (GB11397, dilution 1:500), CDK4 (GB11238-2, dilution 1:100), MDM2 (GB111161, dilution 1:500), CD34 (GB13584, dilution 1:100), Vimentin (GB111308, dilution 1:1000), Leptin (GB11309, dilution 1:600), and HRP-conjugated Goat Anti-Rabbit IgG (GB23303, dilution 1:200). All antibodies were sourced from Servicebio, Wuhan, China. The stained sections were examined using an optical microscope (Optec B302, Chongqing Optec Instrument Co., Ltd., Chongqing, China) equipped with a CCD camera (ICX285A, Sony, Tokyo, Japan).

## 3. Results

**Figure 1:**
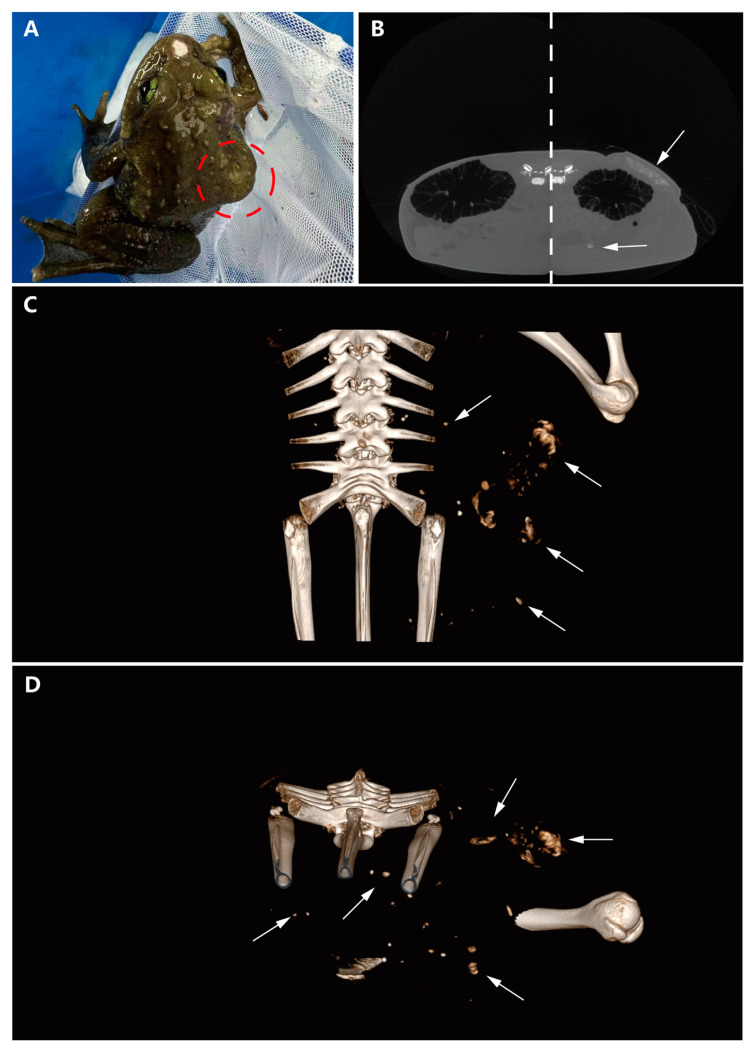
Gross examination and micro-CT scan results. (**A**) Subcutaneous mass visible in the red circle on the right dorsoventral surface. (**B**) CT scan comparison between the diseased and healthy sides (the image is composed of two control images spliced with dotted lines, with white arrows indicating the tumor location). (**C**) 3D model illustrating the spatial relationships of the mass (arrows indicating the tumor). (**D**) Horizontal cross-section of the body cavity revealing dispersed nodular masses (arrows indicating the tumor).

On gross examination, a subcutaneous bulge was observed on the right dorsoventral surface of the frog, along with a mechanical injury at the rostral end (Figure 1A). Micro-CT scans were performed, revealing a region with relatively clear edges but a complex internal structure on the dorsal side of the body cavity, compared to the healthy side. This finding indicated uneven tissue composition in the affected region. Notably, a circular, uneven mass of similar density was also found on the liver (Figure 1B). The spatial relationships of these masses were further illustrated using three-dimensional imaging techniques. The general shape of the largest dorsoventral mass was viewed from the dorsal angle, demonstrating its irregular structure (Figure 1C). Additionally, a cross-sectional image of the body cavity revealed that the nodular mass was dispersed in multiple locations within the cavity, suggesting significant heterogeneity and potential aggressiveness (Figure 1D).

**Figure 2:**
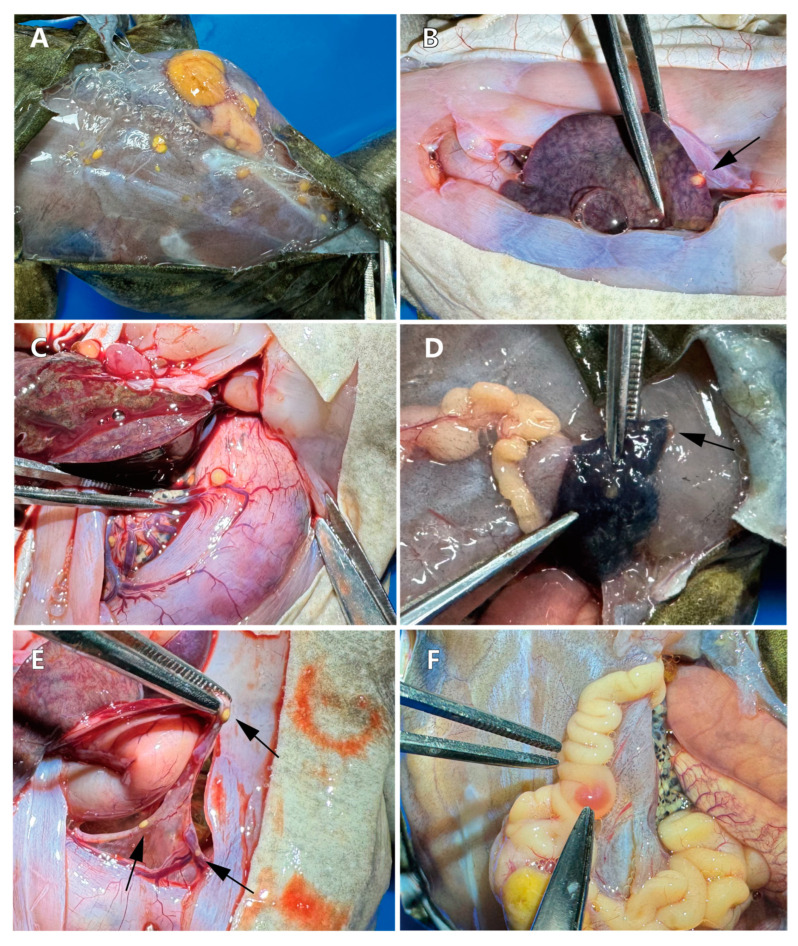
Necropsy and lesion location observation. (**A**) A large yellow mass and multiple diffuse small masses on the back. (**B**) A round yellow mass embedded in the liver (arrow indicates location). (**C**) A yellow mass on the surface of the stomach, with nearby abundant blood vessels. (**D**) Tumor invasion in the right lung (indicated by the arrow). (**E**) Multiple masses observed in the mesentery (arrow indicates location). (**F**) Intestinal lesions and yellow fatty deposits on the intestines.

Dissection from the dorsal side revealed a large mass accompanied by multiple scattered yellow masses, the largest measuring 1.0 × 0.7 × 0.5 cm (Figure 2A). Within the body cavity, a yellow embedded mass was observed in the liver (Figure 2B). Additionally, a yellow mass with a rich blood supply was found on the surface of the stomach (Figure 2C). Similar small tumors were present in the lung adjacent to the larger tumor, though the boundaries of the lung tumors were less distinct compared to those in other locations (Figure 2D). Further examination of the body cavity revealed significant lesions in the mesentery (Figure 2E) and intestines (Figure 2F), all characterized by a predominantly yellow coloration.

**Figure 3:**
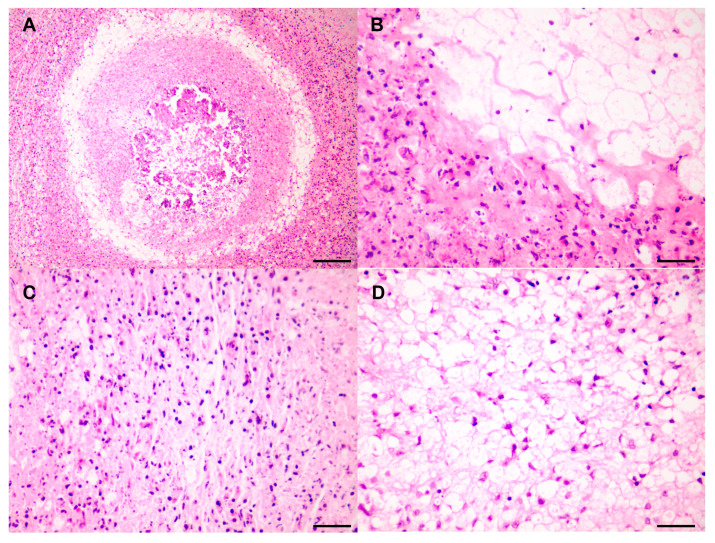
Pathological observation of DDLPS. (**A**) The mass located on the mesentery, observed at low magnification. Scale bar = 120 µm. (**B**) Well–poorly differentiated transition areas. Scale bar = 30 µm. Immunohistochemistry results for (**C**) Poorly differentiated areas. Scale bar = 30 µm. (**D**) Well-differentiated areas. Scale bar = 30 µm.

The following results were observed in the hematoxylin and eosin-stained (H&E) pathological sections. At low magnification (Figure 3A), a central pink area lacking structural organization and showing necrosis is visible, indicating a highly malignant feature characterized by rapid progression. This rapid growth deprives cells in the center of the tumor of adequate blood supply and oxygen, leading to ischemia and necrosis. The high metabolic demands and fast proliferation of the tumor cells are indicative of their aggressiveness and destructive potential [13], suggesting that the tumor cells are highly invasive. The cells surrounding the necrotic area are densely packed, with high cell density and irregular morphology, pointing to dedifferentiated malignant tumor cells. A ring of adipocyte components is observed at the periphery of the high-density cell area, suggesting that the tumor may originate in adipose tissue, which is consistent with the features of dedifferentiated liposarcoma (DDLPS). DDLPS typically presents as a dense mixture of fat cells and undifferentiated tumor cells. At higher magnification, a characteristic transition of components typical of DDLPS is observed, with a clear demarcation between well-differentiated and poorly differentiated areas, marked by an abrupt transition (Figure 3B). In the poorly differentiated region, spindle-shaped fibrous-like structures arranged in a fascicular pattern are visible (Figure 3C). In the well-differentiated area, the cells display significant morphological diversity, with varying nuclear sizes and shapes, and with some nuclei exhibiting thickened chromatin, and prominent nucleoli. The cytoplasm is mostly vacuolated or clear, suggesting these cells might be adipocytes or lipoblasts. Some nuclei are larger and irregular, showing noticeable nuclear morphological changes. Although the cells are generally evenly distributed, some localized areas of increased cell density can be observed (Figure 3D).

**Figure 4:**
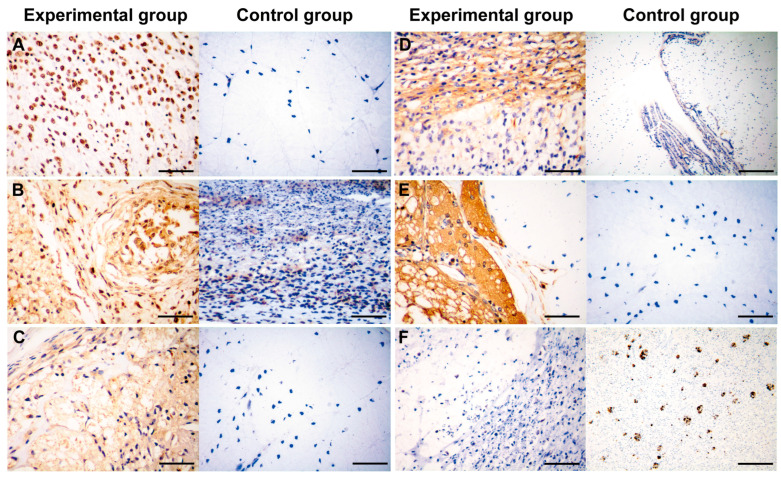
Immunohistochemistry observation of DDLPS. (**A**) S100A4 (+). Scale bar = 30 µm. (**B**) CDK4 (+). Scale bar = 30 µm. (**C**) MDM2 (+). Scale bar = 30 µm. (**D**) CD34 (+). Experimental scale bar = 30 µm, control scale bar = 120 µm. (**E**) Vimentin (+). Scale bar = 30 µm. (**F**) Leptin (−). Scale bar = 30 µm.

Immunohistochemistry was used for differential diagnosis. Tumor cells exhibited a strong nucleolar positive expression of S100A4, with no apparent cytoplasmic staining. The nuclei were diverse, with some being large and irregular (Figure 4A). CDK4 staining showed strong positive expression in both the nuclei and cytoplasm, resulting in a brown coloration of these cellular components. The cells were densely packed, with prominent interstitial spaces in some areas, and the nuclei varied in size and shape, with some being deeply stained and irregular (Figure 4B). MDM2 staining revealed numerous adipocytes, with a dark brown staining of the nuclei and partial cytoplasmic staining (Figure 4C). CD34 expression was significant in both the membrane and cytoplasm of highly differentiated adipocytes and dedifferentiated spindle cells (Figure 4D). Vimentin staining revealed a large number of positive cells, uniformly distributed throughout the tissue, indicating a substantial presence of mesenchymal cells or epithelial–mesenchymal transition (EMT) phenomena. The positive cells exhibited varied morphologies, with some appearing elongated or fibrous, consistent with stromal cells or cells undergoing EMT (Figure 4E). Leptin expression was negative (Figure 4F). The control group was derived from various tissues, including intestinal tissue (S100A4, MDM2, CD34, Vimentin), smooth muscle tissue (CDK4), and liver tissue (Leptin).

## 4. Discussion

According to the 2020 update of the WHO’s Classification of Soft Tissue Tumors, fat cell tumors are categorized into benign, intermediate, and malignant types based on their invasive characteristics. Malignant liposarcomas include well-differentiated liposarcoma, dedifferentiated liposarcoma, pleomorphic liposarcoma, and myxoid liposarcoma [13]. Dedifferentiated liposarcomas (DDLPSs), which account for 18% of all liposarcomas, are characterized by the transformation of well-differentiated liposarcomas (ALT-WDLPS) into non-lipogenic sarcomas. Occasionally, these dedifferentiated components present as pleomorphic liposarcomatoid forms, which are highly aggressive [14].

Diagnosing DDLPS is particularly challenging due to its heterogeneous histological features. DDLPSs often exhibit extensive morphological changes, making it difficult to distinguish them from other liposarcoma types and soft tissue sarcomas [15]. To improve diagnostic accuracy, pathologists commonly use immunohistochemical staining, with MDM2 and CDK4 being key markers for differential diagnosis [10].

In this case, CT imaging and pathological examination revealed that the tumor had clear boundaries but exhibited invasion into surrounding tissues and distal organs, indicating high aggressiveness. Pathological examination further showed metastases and central necrotic areas, which suggest rapid tumor growth and high metabolic activity. The presence of both highly differentiated and dedifferentiated components supports the diagnosis of DDLPS. Additionally, inflammatory cells were observed, indicating a possible immune response to the tumor.

Immunohistochemical analysis demonstrated the positive expression of S100A4, CDK4, MDM2, CD34, and Vimentin, along with the negative expression of Leptin, confirming the tumor’s high aggressiveness and malignancy. These markers are critical for confirming the diagnosis of DDLPS and distinguishing it from other types of fat cell tumors. In particular, the negative expression of Leptin helps differentiate tumor cells from normal adipose tissue, indicating a deviation from normal adipose differentiation pathways [16]. S100A4, a calcium-binding protein associated with cell migration, invasion, and tumor metastasis, indicates high invasive and metastatic potential when expressed [17]. Vimentin, a mesenchymal cell marker, highlights the mesenchymal properties of tumor cells, which are common in tumors of mesenchymal origin [18]. Positive MDM2 expression is often seen in well-differentiated liposarcomas and DDLPS, making it a key diagnostic marker. CDK4, a cell cycle-dependent kinase involved in cell cycle regulation, is frequently used to identify DDLPS. Moreover, CD34, a marker of vascular endothelial cells, indicates the presence of new blood vessels, reflecting the tumor’s angiogenic capacity [19].

Currently, DDLPS is primarily observed in humans, with no known cases in animals. However, the causes of cancer are multifactorial, including genetics, diet, and environmental factors, all of which may contribute to disease development [20]. Amphibians, in particular, are highly sensitive to environmental changes, and the occurrence of liposarcoma in amphibians may signal ecological stress. Studying tumors across species helps researchers understand general tumorigenesis mechanisms and species-specific differences. This comparative approach sheds light on fundamental principles of tumor biology and provides new perspectives and methodologies for human cancer research [21]. By investigating the occurrence and characteristics of liposarcomas in different species, valuable insights can be gained into the underlying mechanisms of the disease. This study not only advances our understanding of veterinary oncology but also provides important insights into amphibian conservation, emphasizing the potential role of environmental stressors in tumor development.

## 5. Conclusions

This study reports the first documented case of dedifferentiated liposarcoma (DDLPS) in veterinary medicine and presents the first pathological evidence of DDLPS in a wild amphibian, *M. medogensis*. These findings are significant for enhancing the understanding of cancer in wildlife species. DDLPS is known for its pronounced histological heterogeneity and invasive potential, which was evident in this case through multiple metastases observed on subcutaneous muscle surfaces and internal organs. The diagnostic process relied primarily on immunohistochemistry, with micro-CT scans, pathological sections, and gross necropsy providing crucial support for a definitive diagnosis.

## Figures and Tables

**Table 1:** Antibody information and retrieval conditions.

Primary Antibody Name	Antigen Retrieval Conditions	Primary Antibody Host Species	Secondary Antibody Name
S100A4	Citrate buffer (pH 6.0), microwave treatment: medium heat for 10 min, pause for 5 min, followed by medium–low heat for 5 min, pause for 2 min, and another round of medium–low heat for 5 min	Rabbit	HRP-conjugated Goat Anti-Rabbit IgG
CDK4	EDTA buffer (pH 9.0), microwave: medium heat 10 min, pause 5 min, medium–low heat 5 min, pause 2 min, medium–low heat 5 min	Rabbit	HRP-conjugated Goat Anti-Rabbit IgG
MDM2	EDTA buffer (pH 8.0), water bath at 90 °C for 30 min	Rabbit	HRP-conjugated Goat Anti-Rabbit IgG
CD34	Citrate buffer (pH 6.0), microwave: medium heat 10 min, pause 5 min, medium–low heat 5 min, pause 2 min, medium–low heat 5 min	Rabbit	HRP-conjugated Goat Anti-Rabbit IgG
Vimentin	EDTA buffer (pH 8.0), water bath at 90 °C for 30 min	Rabbit	HRP-conjugated Goat Anti-Rabbit IgG
Leptin	Citrate buffer (pH 6.0), microwave: medium heat 10 min, pause 5 min, medium–low heat 5 min, pause 2 min, medium–low heat 5 min	Rabbit	HRP-conjugated Goat Anti-Rabbit IgG

## Data Availability

This study did not involve any electronic data. The individual animal specimen from this case was preserved in formalin and stored in the specimen repository of Prof. Jiang at the Chengdu Institute of Biology, along with the histological slides.
